# Correlation of academic emotion and hardiness personality of undergraduate nursing students

**DOI:** 10.1186/s12912-024-01796-1

**Published:** 2024-02-21

**Authors:** Lili Guo, Danfeng Yan, Junping Yan, Rui Jiao

**Affiliations:** 1 School of Nursing, Shanxi University of Chinese Medicine, No. 121 Daxue St., Yuci District, Jinzhong City, 030619 Shanxi China; 2 Department of Psychiatry, Shanxi Mental Health Center, Taiyuan Psychiatric Hospital, School of Mental Health, Shanxi Medical University, Mental Health Hospital of Shanxi Medical University, No. 55 Nanshifang St., Yingze District, Taiyuan City, 030045 Shanxi China; 3 Shanxi Provincial Integrated Traditional Chinese and Western Medical Hospital, Affiliated Hospital of Integrated Traditional Chinese and Western Medicine, Shanxi University of Chinese Medicine, No. 13 Fudong St. Xinhualing District, Taiyuan City, 030013 Shanxi China

**Keywords:** Nursing students, Academic performance, Hardiness personality

## Abstract

**Background:**

Academic emotion is a fundamental emotional concept closely linked to academic achievement. Understanding the connection between academic emotion and the personality trait of hardiness is pivotal in maintaining a stable career orientation throughout one's educational career. Therefore, in pursuit of fostering the robust growth of nursing careers, it is imperative to delve into the academic emotions experienced by undergraduate nursing students. This study endeavors to mitigate the impact of gender differences among nursing students while investigating the intricate relationship between academic emotions and the trait of hardiness in their personalities.

**Methods:**

This study employed a cross-sectional research design. We gathered data from a convenient sample of 292 nursing students enrolled at Shanxi University of Chinese Medicine. Each student provided demographic information and responded to a general academic mood questionnaire, as well as a Hardiness Personality Rating Scale. Subsequently, we used canonical correlation analysis to evaluate the correlation between academic emotion and tenacity personality in 292 undergraduate nursing students.

**Results:**

We discovered that academic emotions among nursing students are predominantly characterized by feelings of disappointment and boredom. Furthermore, personality hardiness is primarily influenced by the dimensions of engagement and control. It is important to note that a heightened level of negative, low-arousal academic emotions can diminish the level of engagement. The first typical correlation coefficient corresponding to academic emotion and hardiness were 0.660. The linear combination of standardized variables of the first typical variable corresponding to academic emotion (X1) = -0.444*negative hyperarousal -0.443 * positive hyperarousal + 0.694 * negative hypoarousal -0.260 * positive hypoarousal. The standardized variable equation of the first typical variable corresponding to hardiness personality (η1) = 0.235* hardiness -0.433* control -0.530* investment -0.303* challenge.

**Conclusions:**

Nursing students generally believe that their input is out of proportion to the return, and this unbalanced emotional experience will seriously affect their academic emotions in China. It is suggested that paying attention to cultivating their tenacious personality traits in the teaching process may help to enhance their academic emotions and enhance the sense of belonging and identity of nursing students engaged in the nursing profession.

## Introduction

Undergraduate nursing students, serving as the future workforce in the field of medical science, require not only proficient professional skills but also resilient and well-rounded personalities for their professional endeavors. This characteristic exerts a profound impact not only on their future careers but also on their college life. Throughout their educational journey, academic emotion emerges as the predominant emotional aspect they encounter. Emotional exhaustion emerges as a significant dimension of academic burnout when assessing its impact on psychological well-being, while resilience plays a crucial role in promoting positive psychological outcomes [1]. Moreover, a strong career belief can contribute to enhancing students' positive academic emotions to some extent. As the old adage suggests, "Interest is the best teacher." However, in the context of higher education in China, major selection often depends on selective testing, and an individual's interests do not solely determine their choice of major. Hence, academic emotion is not solely shaped by students' professional interests. Numerous factors can influence students' academic moods. Research indicates that students' adaptability and engagement positively correlate with academic emotions [2]. Nevertheless, academic emotions are also intertwined with various other factors, including the learning environment, interpersonal relationships, gender, social status, working conditions, and professional identity.

Furthermore, the reciprocal influence between these factors collectively shapes the trajectory of academic emotions. It is worth noting that professional learning experiences can have a profound impact on the development of nursing students' personalities. Notably, hardiness emerges as a pivotal factor contributing to students' success in nursing programs of study [3]. Considering that academic emotion is a recurring emotional response tied to a particular academic setting, it plays a subtle yet influential role in shaping students' knowledge acquisition and personality development. Eventually, we delve deeply into the interplay between hardiness personality traits and academic emotions will be necessary to fully understand all the mechanisms of the academic emotion and hardiness personality, and in turn, the effects on the future careers of nursing students.

## Materials and methods

### Participants

In this study, we focused on nursing students from Shanxi University of Chinese Medicine as our research participants. We used a networking questionnaire tool (Questionnaire Star Software), and the online questionnaire was pushed in the form of WeChat, relying on the platform of a nursing school of Shanxi University of Chinese Medicine from May 1st to May 7th, 2022. An online survey was conducted using a self-administered questionnaire delivered through the Internet. The students who voluntarily participate in the survey. The response rate was 97.3% since 292 out of the 300 distributed survey questionnaires were collected. Given that the nursing profession in our country is typically characterized by a female majority in the last few decades, gender-specific variations may exist in the academic emotions and career aspirations of nursing students. To ensure a balanced representation, the questionnaire distribution was adjusted to include male students in proportion to their presence among nursing students. This approach allowed us to maintain gender balance in our study. The final number of included analyses in the study was 102.

## Instruments

### General academic mood questionnaire for college students

Academic emotion, a concept introduced by the German educational psychologist Pekrun [4], is a central focus of this study. The Chinese version of the General Academic Emotions Questionnaire for college students was compiled by Professor Ma in 2008 [5]. This scale has a Cronaabachs α of 0.641 ~ 0.887 and test–retest reliability of 0.563 ~ 0.886, which serves as our foundation. Building upon the theory of academic emotions, our research explores the diverse emotional experiences associated with students' academic activities during their college years. As research advances, the definition of academic emotions has expanded to encompass reactions to both academic success or failure and emotions experienced during routine homework assignments [6]. The questionnaire comprises 88 self-assessment items, encompassing emotions such as interest, happiness, pride, hope, relief, anger, anxiety, shame, disappointment, and boredom. We employed a five-point scoring system: 5 points for "completely consistent," 4 points for "relatively consistent," 3 points for "uncertain," 2 points for "not quite consistent," and 1 point for "completely inconsistent." The questionnaire exhibited strong internal consistency, with coefficients ranging from 0.641 to 0.887, and satisfactory test–retest reliability, ranging from 0.563 to 0.866. These ten factors within the scale can be classified into four dimensions: the negative hyperarousal dimension encompasses shame, anxiety, and anger. It refers to an elevated state of physiological and psychological arousal, where an individual experiences heightened stress or anxiety levels, often as a response to distressing or traumatic experiences. The positive hyperarousal dimension includes interest, pleasure, and hope. It refers to an elevated state of physiological and psychological arousal, which is often characterized by increased alertness, anxiety, and a heightened stress response. Negative hypoarousal dimensions consist of disappointment and boredom. It is often characterized by feelings of lethargy, lack of motivation, and emotional numbness. The positive hypoarousal dimension comprises pride and relief. Hypoarousal typically refers to a state of reduced physiological and psychological arousal, which is often associated with feelings of lethargy, low energy, and emotional numbness. It is noteworthy that the shaping of college students' personalities may exert a significant influence on their ability to regulate emotions [7, 8].

#### College student hardiness personality rating scale

A hardiness personality is a constructive personality trait that integrates cognitive abilities, behavior, and emotions. It equips individuals with the capacity to maintain a positive outlook, remain optimistic and enterprising, exhibit persistence in both cognitive and emotional aspects, and demonstrate strong self-control when confronted with challenges [9]. A substantial body of research has consistently demonstrated that a hardy personality serves as a valuable resource for effectively managing stress [10, 11]. Professor Lu and colleagues developed the College Students' Hardiness Scale [12], based on the resilience personality theory. This scale established the Chinese resilience personality structure, comprising four dimensions: hardiness, input, control, and challenge. The scale includes 27 items, with a four-point scoring method: "completely in line with" for four points, "in line with" for three points, "somewhat in line with" for two points, and "completely not in conformity with" for one point. The coefficients of the four subscales were 0.785, 0.747, 0.784, and 0.802, respectively, while the total scale exhibited high reliability with a coefficient of 0.910. The hardiness dimension reflects the characteristics of individuals who are resolute and unwavering in their pursuit of goals, maintain an optimistic and enterprising attitude when faced with adversity, and exhibit persistence. The control dimension mirrors the traits of individuals actively managing and influencing the events they encounter, while the engagement dimension signifies individuals' commitment and focus on their activities. Lastly, the challenge dimension represents the qualities of individuals who draw strength from practical experiences, with the total score providing an overall assessment of an individual's degree of personality resilience.

### Data processing

We employed propensity score matching techniques to effectively control for potential confounding factors. Subsequently, we conducted canonical correlation analysis using the Canonical Correlation package within SPSS Statistics for Windows Version 26.0.

## Results

### General academic emotions profile of undergraduate nursing students

We summarized the mean and standard deviation (SD) of general academic emotions among undergraduate nursing students using descriptive statistics. (Table 1).

**Table 1:** General academic mood scale and hardiness personality scales core of college students

Item	Mean ± SD
General Academic Mood Score	
Anxiety	47.59 ± 11.46
Boredom	34.74 ± 11.01
Relief	32.99 ± 6.56
Disappointment	27.56 ± 7.58
Pride	31.24 ± 5.86
Shame	23.89 ± 5.42
Happy	26.17 ± 4.83
Hope	27.71 ± 5.21
Angry	15.16 ± 3.94
Interesting	18.03 ± 3.58
Negative hyperarousal	86.64 ± 19.04
Positive hyperarousal	71.91 ± 12.89
Negative hypoarousal	62.30 ± 18.01
Positive hypoarousal	64.23 ± 11.86
Hardiness Personality Score	
Hardinessness dimension	15.41 ± 3.65
Control dimension	21.45 ± 5.23
Engagement dimension	15.83 ± 3.86
Challenge dimension	18.31 ± 4.59
Total score	71.00 ± 16.47

### Overview of the hardiness personality of college students

The distribution of mean and standard deviation values for the hardiness personality traits among undergraduate nursing students. (Table 1).

### Correlation analysis of undergraduate academic emotions and hardiness personality

We grouped ten indicators of undergraduate academic emotions as one set of variables, and the four factors from the hardiness personality scale as another set of variables. Subsequently, we conducted canonical correlation analysis, resulting in four canonical correlation variables (Table 2). Notably, the first canonical variable reached a significant level (*P* < 0.001) upon testing.

**Table 2:** Typical variables and tests of general academic emotions and hardiness personality of undergraduate nursing students

No	Canonical correlation coefficient	Wilk ‘s	F	df	P
1	0.624	0.548	4.328	16.000	*P* < .001
2	0.294	0.897	1.294	9.000	*P* = 0.240
3	0.120	0.982	0.487	4.000	*P* = 0.745
4	0.062	0.996	0.412	1.000	*P* = 0.523

**Fig. 1:**
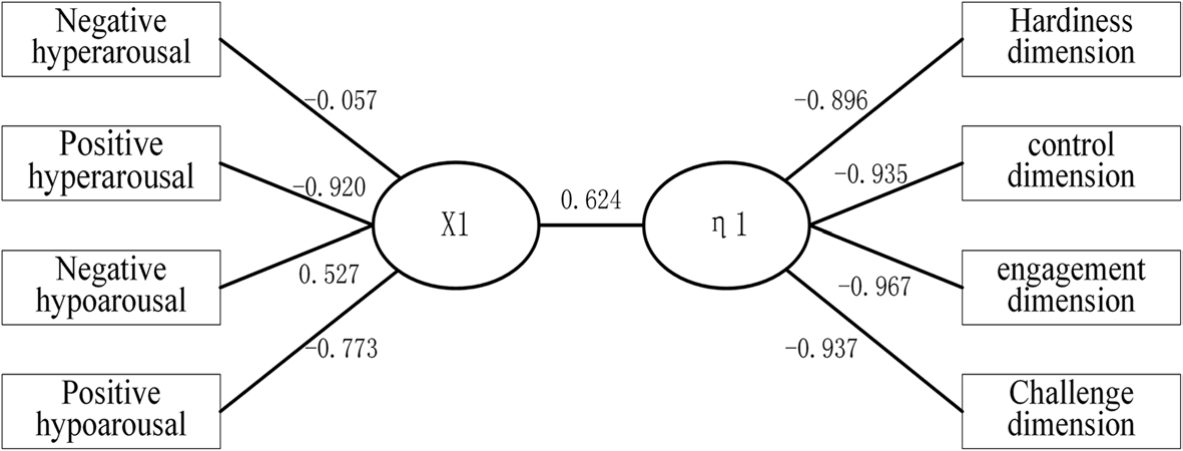
A canonical correlation analysis between hardiness personality and academic emotion

Based on the standardized canonical coefficient (Table 3), the first canonical variable, X1, can be expressed as follows: -0.444* negative hyperarousal -0.443* positive hyperarousal + 0.694* negative hypoarousal -0.260* positive hypoarousal. It is evident that the negative hypoarousal dimension carries the most significant weight, indicating that this dimension primarily influences the typical variables associated with academic emotions among undergraduate nursing students. Turning to the first typical variable of hardiness personality traits (η1) (Table 4), it can be represented as: 0.235* hardiness dimension -0.433* control dimension -0.530* investment dimension -0.303* challenge dimension. Notably, the investment dimension exhibits the highest coefficient among personality traits, followed by the control dimension. This observation underscores that the levels of investment and control dimensions predominantly determine the typical variables associated with a hardiness personality. Consequently, we performed a canonical correlation analysis between hardiness personality and academic emotion (Fig. 1). The inverse relationship between the degree of low arousal and the investment dimension in both variables suggests that a negative low arousal degree of academic emotion may indeed weaken the level of investment, aligning with the objective reality.

**Table 3:** Standardized typical coefficient and correlation coefficient (load) of each factor of general academic emotion of undergraduate nursing students

Variables	The standardized canonical correlation coefficient				Correlation coefficient load			
	X 1	X2	X3	X4	X1	X2	X3	X4
Negative hyperarousal	-0.444	-0.094	0.132	1.366	-0.057	-0.573	-0.781	0.241
Positive hyperarousal	-0.443	-2.002	-0.539	-2.081	-0.920	0.059	-0.330	-0.202
Negative hypoarousal	0.694	0.909	-1.072	-1.149	0.527	-0.143	-0.831	0.109
Positive hypoarousal	-0.260	0.829	-.098	1.594	-0.773	0.465	-0.360	0.237

**Table 4:** Typical and correlation coefficient of the standardized hardiness personality of undergraduate nursing students (Load)

Variables (dimension)	The standardized canonical correlation coefficient				Correlation coefficient load			
	η1	η2	η3	η4	η1	η2	η3	η4
Hardiness	0.235	0.167	-1.433	2.453	-0.896	0.018	-0.325	0.302
Control	-0.433	-2.002	0.242	-0.935	-0.935	-0.323	-0.145	0.027
Engagement	-0.530	0.625	2.156	0.710	-0.967	0.156	0.129	0.157
Challenge	-0.303	1.192	-1.095	-2.146	-0.937	0.212	-0.266	-0.080

## Discussion

The relationship between academic emotions and hardiness personality in college students differs depending on the professional. Therefore, it is necessary to adopt specific training according to the situation of college students' choice, which is conducive to college students' happy learning, cultivation of perfect personality, and improvement of training quality. The academic emotions of nursing students may be related to the difference in gender [13]. However, the influence of stress on academic emotions during a stressful task can be reduced by cultivating nursing students' interest and increasing their motivation to learn nursing skills [14, 15]. Weixiao et al. conducted a cross-sectional survey. Males had higher positive and negative hyperarousal emotions than females in a study of 258 nursing undergraduates, and males experienced more positive emotions than females. Research has discovered that emotional interventions can improve emotional regulation strategies that may help to reduce perceived psychological stress in nursing students [16]. In this study, the academic emotion of undergraduate nursing students exhibited a negative tendency of low arousal under the premise of excluding the influence of gender through the method of gender orientation matching. Previous studies mentioned that Chinese nursing students experienced more negative academic emotions may be related to a low professional identity. These findings emphasize the potential benefits of implementing emotional support and interventions within nursing education programs to enhance students' well-being and academic success.

Positive emotions play a pivotal role in enhancing the nursing competence of students [14, 17]. Various researchers have proposed that nursing students benefit from exposure to challenging situations that draw upon their resilience and determination to overcome difficulties in the future [18]. In the realm of nursing education, it is crucial to focus on not only imparting professional skills but also nurturing and developing essential personality traits. The concept of hardiness personality traits, serving as a protective factor, assumes particular significance. These traits can significantly enhance the capacity of nursing professionals to adapt to and effectively manage stress [19]. As such, a comprehensive approach to nursing education should encompass the cultivation of both technical proficiency and the fortification of character traits that enable individuals to thrive in the demanding healthcare environment. This study has unveiled that engagement and control stand as the principal factors influencing negative low-arousal emotions. For nursing students, honing their capacity to manage pressure within the framework of a resilient personality is integral to their readiness for the demanding role of professional nurses in practical healthcare settings [20]. Moreover, considering the disparities in academic emotions between nursing students and those in other disciplines, it becomes paramount to bolster their sense of mission, honor, and professional identity. Such efforts can effectively mitigate the adverse impact of negative hypoarousal emotions [21]. Numerous investigations have underscored the positive impact of emotions on medical students' academic performance, thereby indirectly influencing their overall academic emotional well-being [6, 22]. Additionally, a profound understanding of students' academic emotions and the ability to stimulate a comprehensive range of academic emotions hold significant implications for nurse educators when devising course content [23]. Furthermore, it is imperative to create a conducive learning environment that encompasses a favorable atmosphere and elevates the arousal levels of academic emotions. This approach contributes significantly to enhancing students' sense of belonging and concentration within their chosen field of study. In the context of nursing education, particular attention should be directed toward the cultivation of hardiness traits. This focus is instrumental in fostering a resilient and contented nursing workforce, thereby promoting the efficacy of nursing career education. Professional nursing educators bear the responsibility of nurturing these qualities to ensure a stable and satisfied cadre of nursing professionals.

## Conclusions

In conclusion, we find that there is a mismatch between nursing students' efforts in academic pursuit and their perception of rewards, which results in negative academic emotions with low arousal. Therefore, incorporating this easily accessible measurement of academic emotion and cold-resistant personality into their educational strategies may help to enhance the professional honor of nursing students and stabilize their confidence in the nursing profession.

## Data Availability

All relevant data are included with in the manuscript document. If necessary, it is possible to contact the corresponding author to obtain additional materials.

## Abbreviations

M: Mean
SD: Standard deviation
